# Pathogenicity and Virulence of *Trueperella pyogenes*: A Review

**DOI:** 10.3390/ijms20112737

**Published:** 2019-06-04

**Authors:** Magdalena Rzewuska, Ewelina Kwiecień, Dorota Chrobak-Chmiel, Magdalena Kizerwetter-Świda, Ilona Stefańska, Małgorzata Gieryńska

**Affiliations:** Department of Preclinical Sciences, Faculty of Veterinary Medicine, Warsaw University of Life Sciences, Ciszewskiego 8 St., 02-786 Warsaw, Poland; ewelina1708@gmail.com (E.K.); dorota.chrobak@wp.pl (D.C.-C.); magdakiz@wp.pl (M.K.-Ś.); i.stefanska@gmail.com (I.S.); malgorzata_gierynska@sggw.pl (M.G.)

**Keywords:** *Trueperella pyogenes*, virulence, pyolysin, infection, pathogenicity, immune response, *Actinomycetales*

## Abstract

Bacteria from the species *Trueperella pyogenes* are a part of the biota of skin and mucous membranes of the upper respiratory, gastrointestinal, or urogenital tracts of animals, but also, opportunistic pathogens. *T. pyogenes* causes a variety of purulent infections, such as metritis, mastitis, pneumonia, and abscesses, which, in livestock breeding, generate significant economic losses. Although this species has been known for a long time, many questions concerning the mechanisms of infection pathogenesis, as well as reservoirs and routes of transmission of bacteria, remain poorly understood. Pyolysin is a major known virulence factor of *T. pyogenes* that belongs to the family of cholesterol-dependent cytolysins. Its cytolytic activity is associated with transmembrane pore formation. Other putative virulence factors, including neuraminidases, extracellular matrix-binding proteins, fimbriae, and biofilm formation ability, contribute to the adhesion and colonization of the host tissues. However, data about the pathogen–host interactions that may be involved in the development of *T. pyogenes* infection are still limited. The aim of this review is to present the current knowledge about the pathogenic potential and virulence of *T. pyogenes*.

## 1. Introduction

**Figure 1:**
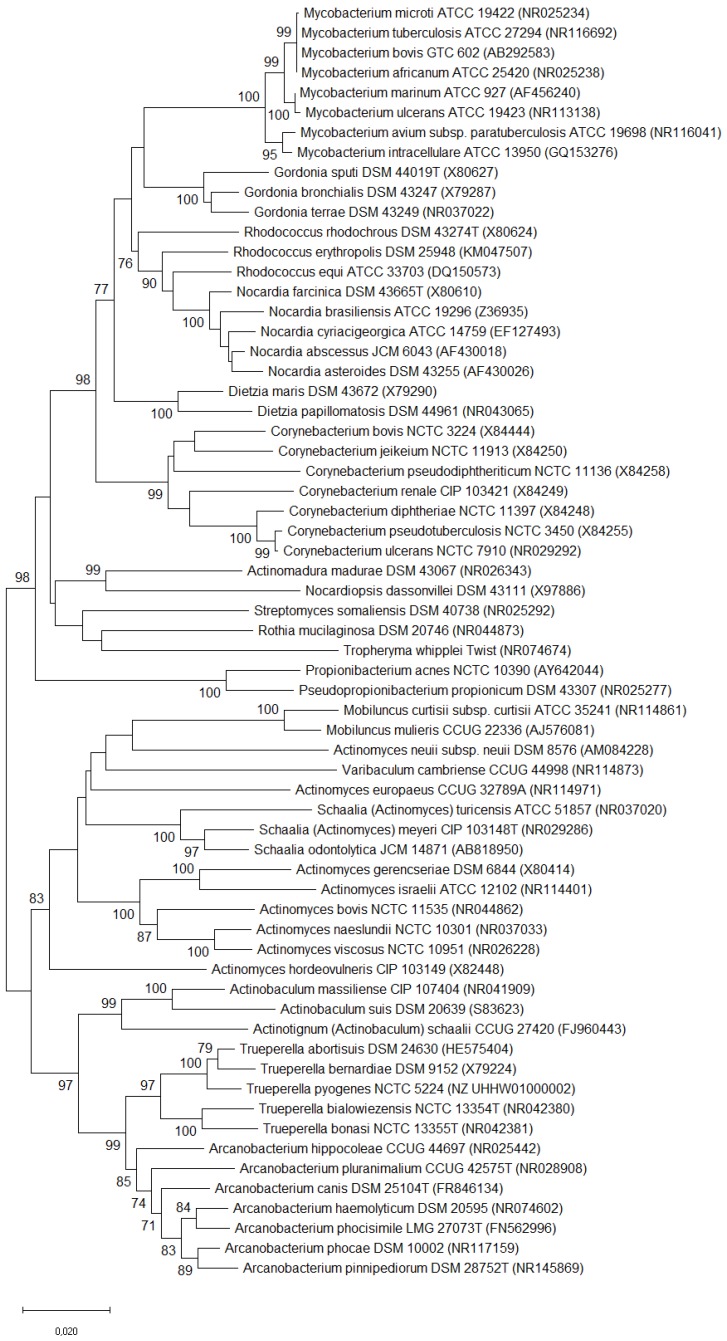
Neighbor-joining [15,16] phylogenetic tree based on 16S rRNA gene sequences (a total of 1422 positions in the final dataset) of *Trueperella pyogenes*, other *Trueperella* species, and related taxa. Only bootstrap values of 70%, based on 1000 replicates, are shown next to the branches [17]. The scale bar represents the number of substitutions per site. The analysis involved 64 nucleotide sequences derived from the GenBank^®^ database, and the evolutionary relationships were calculated in MEGA X [18].

The species *Trueperella pyogenes* [1], previously classified as *Arcanobacterium pyogenes* [2], *Actinomyces pyogenes* [3,4], and formerly as *Corynebacterium pyogenes* [5], belongs to the family *Actinomycetaceae*, in the order *Actinomycetales* of the class *Actinobacteria*, the so-called actinomycetes [6]. A dendrogram representing the phylogenetic relationship between this bacterium and some other pathogenic *Actinomycetales* is shown in Figure 1. *T. pyogenes* is a Gram-positive, pleomorphic, non-spore-forming, non-motile, non-capsulated, facultatively anaerobic rod, which is characterized by a fermentative metabolism and strong proteolytic activity [4]. Its growth requirements are not excessive, but media enriched with blood or serum need to be used for the culture. The preliminary recognition of *T. pyogenes* isolates is based on the cell morphology; the features of colonies, which are surrounded by a zone of beta-haemolysis on blood agar; and a negative catalase assay. Then, the biochemical properties can be tested for species determination [7]. Sometimes, additional bacteriological methods other than the conventional ones are necessary for the differentiation and appropriate identification of isolates. New techniques, such as loop-mediated isothermal amplification (LAMP) assay, matrix-assisted laser desorption/ionization time-of-flight (MALDI-TOF) mass spectrometry, Fourier transform infrared (FT-IR) spectroscopy, or 16S rRNA gene sequencing may be useful for the diagnostics of *T. pyogenes* infections [8,9,10,11,12,13]. Those methods enable the recognition of the closely related taxa of the order *Actinomycetales*, and sometimes the reclassification of some of them [14].

*T. pyogenes* is considered to be a part of the biota of skin and mucous membranes of the upper respiratory and urogenital tracts of animals [19,20,21]. Moreover, this bacterium was also isolated from the wall of bovine rumen and swine stomachs, as a gastrointestinal microbiota [22,23], and from the udders of clinically healthy cows [24]. However, *T. pyogenes* is also an important opportunistic pathogen. This species, like other well-known actinomycetes, such as *Corynebacterium pseudotuberculosis* or *Rhodococcus equi*, is an etiological agent of common suppurative infections in animals. In the case of *C. pseudotuberculosis*, the highest susceptibility to the infection is observed in small ruminants, and abscesses are located mainly in the lymph nodes [25,26]. *R. equi* is a cause of pyogenic infections, mostly in horses, and lesions are found mainly in the respiratory tract [27,28]. Whereas *T. pyogenes* is pathogenic for a variety of animal species, and purulent or necrotic lesions may occur in different host tissues. Interestingly, there were no relationships found between the virulence gene profiles of *T. pyogenes* strains and their origin, a type of infection and a host [13,21,29,30,31]. However, Ashrafi Tamai et al. [32] reported a significant association between the virulence genotypes and clonal types of *T. pyogenes* isolates, and the severity of the clinical symptoms in postpartum cows with metritis.

Although *T. pyogenes* is a bacterium that has been known of for a long time, many questions concerning the mechanisms of infection pathogenesis, as well as its reservoirs and routes of transmission, still remain poorly understood. The aim of this review is to present the current knowledge about the pathogenicity and virulence potential of this opportunistic animal pathogen.

## 2. Pathogenicity

*T. pyogenes* infections occur in both domestic and wild animals worldwide, but are rare in humans. The prevalence of *T. pyogenes* isolation may differ, depending on a host species and a geographic region. The majority of published data concerns *T. pyogenes* infections in food animals and comes from Europe [21,33,34,35,36,37,38], China [39,40,41,42,43], Japan [44,45], Brazil [30,46], and the United States [47,48,49,50]. In livestock, the diseases caused by *T. pyogenes* generate significant economic losses, mainly in cattle and swine breeding, causing a reduction of meat and milk yield, as well as decreased reproductive efficiency and sometimes the necessity to cull diseased animals. The clinical course of these suppurative infections may be severe, with different mortality rates, which increase in the case of misdiagnosis or inappropriate treatment. Beta-lactams, tetracyclines, and macrolides are the antibiotics most often used to treat *T. pyogenes* infections. A question of the therapy efficacy is especially important, as the antimicrobial resistance in *T. pyogenes* becomes an emerging problem because of the common use of these drugs in agriculture [29,32,34,39,40,42,44,47,49,51,52].

Generally, the infections caused by *T. pyogenes* have an opportunistic nature, in which adverse environmental and host-related factors play a relevant role in the disease establishment [45,50,53,54]. Nevertheless, the risk factors of the infection development are sometimes difficult to estimate. Curiously, there are no obvious differences observed between the virulence genotypes of commensal and clinical *T. pyogenes* isolates, although, in some investigations, a gene encoding one of the virulence determinants, fimbria A, was found more frequently in the isolates obtained from infected cows than from healthy ones [49,55]. However, the in vitro study of Ibrahim et al. [56] showed significant differences in the expression level of eight known virulence genes in *T. pyogenes* isolated from the uterus of a cow with clinical endometritis, and the isolate from the uterus of a healthy cow. This indicates the importance of the regulatory mechanisms of the virulence gene expression in the infection development, which can be also demonstrated in an example of the pyolysin gene expression [57]. In other study, a relationship between the clonal types of isolates and their origin, from a diseased or healthy host, was noted [21]. All of these findings suggest that other unknown bacterial factors may also contribute to the establishment and development of the *T. pyogenes* infection.

*T. pyogenes* may cause infection as a primary etiological agent, but more frequently, this species is involved in polymicrobial diseases, such as mastitis [45], uterine infections [55], interdigital phlegmon [58], or liver abscesses [59]. This bacterium may be recovered from a mix infection of various bacterial species, but especially frequently with Gram-negative anaerobes, such as *Fusobacterium necrophorum*, *Bacteroides* spp., or *Peptoniphilus* (formerly *Peptostreptococcus*) *indolicus*. In these cases, purulent and necrotic lesions are usually observed, leading to systemic signs and resulting in animal death. A particularly strong synergistic interaction occurs between *T. pyogenes* and *F. necrophorum* [60]. It consists in the stimulation of *F. necrophorum* growth by a diffusible and heat-labile product of *T. pyogenes*, which probably decreases the oxygen pressure and the oxidation-reduction potential in a site of infection, generating conditions advantageous for this anaerobe [61]. On the other hand, a leukotoxin produced by *F. necrophorum* protects *T. pyogenes* against phagocytosis, because of its ability to lysis leukocytes or to induce their apoptosis, depending on its concentration [59]. Moreover, lactic acid, which is a metabolic product of *T. pyogenes*, can be used by *F. necrophorum* as an energy substrate. Other bacteria, especially *Escherichia coli*, are also often associated with *T. pyogenes* co-infections, mostly postpartum uterine infections [55,62,63]. However, the synergistic effect between both of these bacteria is not evident. The findings of Zhao et al. [64] indicated that *N*-acyl homoserine lactones from *E. coli* and *Pseudomonas aeruginosa*, which act as the quorum-sensing (QS) signal molecules, can inhibit the growth and virulence of *T. pyogenes* in vitro. The recent in vivo study of Huang et al. [65], conducted in a mouse model, confirmed these observations.

### 2.1. T. pyogenes Infections in Animals

In livestock, *T. pyogenes* infections occur mainly in cattle, swine, sheep, and goats, rarely in horses or birds, and are often associated with heavy economic losses.

In cattle, *T. pyogenes* mainly causes infections of the reproductive tract and the mammary gland, as well as pneumonia and liver abscessation. The most prevalent diseases in dairy cows related with this bacterium are metritis and endometritis, which may develop in a clinical form in about 23–52% of animals after parturition [29,32,38,39,66]. *T. pyogenes* together with many other bacteria comprises a vaginal biota of healthy cows [67], and may also colonize and persist in the uterus of dairy cows with normal puerperium [21,38]. However, as an opportunistic pathogen, this bacterium can invade the distant parts of the reproductive tract, especially after parturition, when the protective epithelium of the endometrium is disrupted, and it can also increase the influx of inflammatory cells in these tissues [50,68]. The presence of *T. pyogenes* in the endometrium is correlated with the damage of the tissue because of the cytolytic activity of the pyolysin against the endometrial stromal cells, which are particularly sensitive to this cholesterol-dependent toxin [68,69]. The ability of *T. pyogenes* to produce inflammatory lesions in the endometrium was confirmed by the findings of Lima et al. [70]. They noted that after the intrauterine infusion of *T. pyogenes* suspension containing 10^9^ colony-forming units/mL, moderate to severe endometrial inflammation developed in the studied cows, and additionally, premature luteolysis was observed in some animals. The uterine inflammation, usually later postpartum, associated with *T. pyogenes*, has a form of subclinical or clinical endometritis, and may reduce the reproductive performance and milk yield [63,71,72]. Bonnett et al. [73] noted that cows with *T. pyogenes* infection took significantly longer to conceive. Moreover, the study of Boer et al. [63] demonstrated that *T. pyogenes* isolation at day 21 postpartum was associated with the subsequent diagnosis of purulent vaginal discharge, and this observation was confirmed by Sheldon et al. [74]. *T. pyogenes* can also have a lethal impact on the oviductal epithelial cells, as it was demonstrated by Mesgaran et al. [75] in the in vitro study. In general, in the cows infected by *T. pyogenes*, an increased prevalence of clinical endometritis is observed [50]. Frequently, uterine disorders are from co-infections with *T. pyogenes* and other bacteria, such as *E. coli*, *Streptococcus* spp., *Staphylococcus* spp., *Fusobacterium* spp., *Prevotella* spp., and *Clostridium* spp. [39,55,62,71,76,77,78]. Such polymicrobial uterine infections, especially those with anaerobes, result in an increased purulent secretion and higher severity of lesions [76,79]. The problems regarding parturition, the subsequent negative energy balance, or hyperketonemia are considered to be important risk factors for these diseases [63,80].

Another important and common *T. pyogenes* infection in cattle is mastitis, which may affect lactating and dry cows, as well as heifers [30,37,43,45,46,81,82,83,84]. *T. pyogenes* is well known as one of the crucial agents of polymicrobial infection, called summer mastitis, which occurs mainly in pastured cows during the summer, and is associated with pathogen transmission by an insect, *Hydrotaea irritans* [85,86]. However, Madsen et al. [87] demonstrated no differences in the rates of *T. pyogenes* isolation from the mastitis cases in stabled and pastured cattle. Similarly, Ribeiro et al. [46] and Ishiyama et al. [45] did not observe a seasonality of the *T. pyogenes* mastitis occurrence during their long-term survey of the disease. Moreover, it was reported that *T. pyogenes* alone can cause clinical mastitis called pyogenes mastitis, even with a high severity of symptoms [45,85]. The mammary gland inflammation caused by this bacterium is characterized by severe pyogenic lesions in the mammary tissue, and malodorous and purulent milk, especially in case of co-infection with anaerobes, decreasing the milk yield and the low recovery rate [45]. The anaerobes most often involved in mastitis together with *T. pyogenes* are *P. indolicus*, *F. necrophorum*, and *Prevotella melaninogenica* (formerly *Bacteroides melaninogenicus*). All of these bacteria can colonize the mucous membranes and skin of clinically healthy cattle, but *P. indolicus* and *T. pyogenes* were the most frequently isolated from a teat skin [88]. Regardless of the many investigations on *T. pyogenes* mastitis, little is still known about the factors involved in the establishment and persistence of that infection.

*T. pyogenes* also contributes to many other disorders in cattle, among them, liver abscesses and interdigital phlegmon have a more significant economic impact. Those infections are mixed with anaerobes, mainly *F. necrophorum*, characterized by the synergistic interaction of both of the bacteria mentioned previously [59]. Liver abscesses related to *T. pyogenes* infection occur mostly in feedlot cattle, with a varying frequency from 2% to 80% [59,89,90,91]. The infection is associated with *T. pyogenes* presence in the rumen wall, in which the primary lesions can form and then bacteria can penetrate by a hepatic portal venous system to a liver, where abscesses form as secondary infection foci [60]. In the case of abscesses rupturing, systemic signs can develop, resulting in animal death. The development of liver abscesses is probably associated with ruminal acute or subacute acidosis, which can induce primary damage in the protective surface of the rumen wall.

Bovine interdigital phlegmon incidence in dairy cows is usually 2–5% per lactation, but some cases of outbreaks were also reported [35,58,92,93]. The role of *T. pyogenes* in the pathogenesis of this polymicrobial disease is not well defined. Kontturi et al. [58] reported that this bacterium is rather a secondary pathogen associated with the healing stage of the infection, while Bay et al. [94] did not detect *T. pyogenes* at all in the interdigital phlegmon investigation using 16S rRNA gene sequencing.

Moreover, *T. pyogenes* may cause a variety of other purulent infections in cattle such as pneumonia, encephalitis, pyelonephritis and kidney abscesses, lymphadenitis, endocarditis, and abscesses of various localization [30,31,36,44,46,95,96]. This pathogen was also isolated from cases of septicaemia and abortion [36,44,46,97].

In swine, *T. pyogenes* is a common agent of pneumonia, pleuritis, endocarditis, osteoarthritis, polyarthritis, mastitis, reproductive tract infections, and septicaemia [13,29,35,44,98,99,100,101,102]. Abscesses—superficial, muscular, or located in different organs—occur frequently, and may lead to the development of systemic purulent infection and inflammation of lungs, liver, kidneys, muscles, bones, joints, or other tissues [13,46,103]. In many cases, these are infection mixed with different microorganisms, as is observed in *T. pyogenes* infections in cattle. Diseases of swine associated with *T. pyogenes* are an emerging clinical, epidemiological, and economic problem, because they usually result in the necessity for the elimination of infected animals from a herd, and the discard of carcasses with suppurative lesions at slaughterhouses [103]. It has been reported that a number of factors can predispose to the development of these disorders, among other viral infections, that cause immunosuppression, for example with porcine reproductive and respiratory syndrome virus (PRRSV) [104].

In small ruminants, *T. pyogenes* is mostly a cause of abscesses formation in different tissues and localized in various parts of the body, including bone marrow and foot (footrot) abscesses [29,31,105,106,107,108,109,110]. Those lesions differ from that observed in the case of caseous lymphadenitis caused by *C. pseudotuberculosis* [111]. Moreover, *T. pyogenes* can be associated with purulent, mainly polymicrobial disorders in sheep and goats, such as pneumonia, lymphadenitis, arthritis, osteomyelitis, reproductive tract infections, mastitis, and septicaemia [30,35,36,46,112,113,114].

In other domestic animals, infections related to *T. pyogenes* are rare. It may be connected with the fact that this bacterium does not belong to their normal biota. Therefore, there are only a few data on the occurrence of *T. pyogenes* infections in companion animals. The first reported cases referred to otitis externa in a cat and cystitis in a dog [115], lung abscess in a dog [116], and feline pyothorax [117]. Moreover, *T. pyogenes* was isolated from cases of wound infection, abscesses, vaginitis, pneumonia, and encephalitis in dogs [30,35,36,46]. Recently, an interesting case of *T. pyogenes* and *Brucella abortus* co-infection in a cat and a dog was described by Wareth et al. [118]. Both animals lived on a dairy cattle farm, where cases of abortion and mastitis in cows were noted. The bacteria were isolated in a mixed culture from the uterine discharge of a bitch after abortion and a cat with pyometra.

Infections related to *T. pyogenes* were noted sporadically in horses, and included single cases of metritis, orchitis, mastitis, septicaemia, umbilical infection in foals, abscesses, and wound infection [30,36,46,119].

Furthermore, two cases of suppurative disorders in rabbits caused by *T. pyogenes* were published. In the first case, this bacterium was isolated from lung lesions, and in the second one, from necrotic foci in liver, spleen, lung, and brain [36,120].

The incidence of *T. pyogenes* infections in birds seems to be very low. Some cases of clinical lameness and osteomyelitis in turkeys were reported [121,122]. A unique case of liver abscesses in pigeons was described by Priya et al. [123].

In wildlife, *T. pyogenes* may be associated with many types of purulent disorders occurring in free-living and captive animals. *T. pyogenes* infections were reported the most frequently in ruminants and other herbivores, in which the bacteria were also found as a resident microbiota of the skin and mucous membranes of respiratory and urogenital tracts [20,54,124].

In the United States and Canada, *T. pyogenes* is an emerging pathogen of captive or free-ranging white-tailed deer (*Odocoileus virginianus*), which cause intracranial abscessation—suppurative meningoencephalitis disease complex (the intracranial abscess disease) that occurs with a variable frequency, depending on animal populations [54,124,125,126,127]. *T. pyogenes* may be also involved in pneumonia and necrobacillosis occurring in this animal species [128,129].

Furthermore, *T. pyogenes* was also isolated as an etiological agent of chronic purulent infections, including keratoconjunctivitis, brain and foot abscesses, in other cervids, such as Key deer (*Odocoileus virginianus clavium*) [130], fallow deer (*Dama dama*) [53], roebuck (*Capreolus capreolus*) [131], red deer (*Cervus elaphus*) [132], and mule deer (*Odocoileus hemionus*) [133]. *T. pyogenes* is a prevalent cause of variably located abscesses in forest musk deer (*Moschus berezovskii*), noted mainly in farm animals [40,134].

In European bison (*Bison bonasus*), *T. pyogenes* is associated with abscesses of the liver, spleen, lungs, lymph nodes, skin, and especially with urogenital tract infections [135]. In female bison, abscesses of various localizations were observed. In male bison, *T. pyogenes* is considered to be one of the etiological agents of a chronic necrotizing and ulcerative inflammation of the prepuce and penis (balanoposthitis) [135].

Additionally, *T. pyogenes* infections were reported in antelopes [29,136,137], an okapi (*Okapia Johnstoni*) [138], bison (*Bison bison*) [139], Bighorn sheep (*Ovis canadensis*) [140], a chamois (*Rupicapra pyrenaica*) [112], camels [141,142,143,144], an elk [36], and reindeer [145,146]. Moreover, some other sporadic cases of infectious diseases associated with *T. pyogenes* were described in Grey Slender lorises (*Loris lydekkerianus nordicus*) [147,148], macaws, and elephants [48], as well as in reptiles, a bearded dragon, and a gecko [149].

### 2.2. T. pyogenes Infections in Humans

Infections caused by *T. pyogenes* in humans are sporadic, mostly occur in immunosuppressed patients, and are connected with occupational exposure, especially relating to contact with farm animals and their environment [150]. Published data concerning *T. pyogenes* infections in humans are limited, and include, among others, reports on endocarditis [151,152,153,154,155], endemic leg ulcers [156,157], pneumonia [158], arthritis [159,160], sepsis [161], and various purulent lesions and abscesses [162]. As *T. pyogenes* was never demonstrated as a commensal microbiota of humans, those infections should be considered as zoonotic diseases [48]. Although, a probability of animal-to-human transmission of this pathogen has not been well estimated and confirmed.

## 3. Reservoir, Transmission, and Routes of Infection

Most actinomycetes are widespread in the natural environment, being found in various ecological niches as saprophytes, but they may also constitute a commensal biota of humans and animals, and some of them can be opportunistic pathogens [163,164]. Curiously, there is no published characterization of environmental *T. pyogenes* isolates, for example from soil, which might be considered non-pathogenic, as it is known, for instance, in case of environmental strains of *R. equi*, a typical soil opportunistic pathogen [165]. Although there are no data on *T. pyogenes* ability to replicate in soil or water, certainly, it can persist for some period in such environmental conditions. For example, *C. pseudotuberculosis*, the related actinomycete, can survive for several weeks in the environment [166]. Considering the nutritional requirements of *T. pyogenes*, it should be assumed that the bacterium can replicate only in the environment rich in peptides, fermentable carbohydrates, inositol, and hemin, and at the temperature range of 20 to 40 °C [4]. Therefore, it seems that a main reservoir and source of this bacterium are animals of various species.

Little is known about the dissemination of *T. pyogenes* infections, and about the transmission of the pathogen. It is suggested that the majority of infections have an endogenous character, as the bacteria are a common component of the skin and mucous membrane biota [48]. However, the possibility of exogenous infections should also be considered, because bacteria may be transmitted, for instance, by contaminated husbandry utensils and equipment, or directly from animal to animal [30,46]. The natural environment contaminated by *T. pyogenes* is suggested to be an important source of the bacteria, especially in the case of mammary or foot disorders in domestic and wild animals [46,53,109]. In addition, climate conditions, such as a high humidity and mild temperature, are factors favouring the infection occurrence [53]. In the case of summer mastitis, *T. pyogenes*, exceptionally, may be transmitted between animals by biting flies [86]. However, a possibility of tissue penetration by these bacteria through a micro-trauma caused by ectoparasites, such as ticks, should be also considered. The preliminary results of the study on ticks collected from the skin of European bison indicated a potential contribution of those arthropods to *T. pyogenes* transmission [167].

The observations from different studies indicate a potential threat of *T. pyogenes* transmission from wild to domestic animals, or the other way around [53,135]. It is very possible, regarding the frequent co-occurrence of wildlife species and livestock animals on the same agricultural areas, such as pastures and meadows, which can serve as a reservoir for this pathogen.

The data on the genetic relationship of *T. pyogenes* isolates occurring in a particular host population, and on the dissemination of strains amongst animals and the environment are lacking. The results of a few epidemiological studies on bovine and swine isolates are available [10,13,21,32,148]. In some of them, an association between the clonal types and the development of clinical infection by a strain was shown [21,32,148], while in other investigations, such a correlation was not found [10,13]. A variety of molecular methods, characterized by various discriminatory powers, were used for *T. pyogenes* differentiation, which enabled the phylogenetic analysis of strains to be performed, for example the 16S rRNA gene sequencing [135,149], single enzyme amplified fragments length polymorphism (SE-AFLP) [13], BOX-PCR [21,32], the superoxide dismutase A gene (*sodA*) sequencing [36], random amplification polymorphic DNA (RAPD-PCR), and multilocus sequence analysis (MLSA) [148]. The usefulness of some of them was proved by Nagib et al. [148] during the study on *T. pyogenes* isolates from lorises, which showed a close relationship, indicating their clonal origin. Unfortunately, the gold standard method used for genomic fingerprinting, pulse field gel electrophoresis (PFGE), was never reported as a technique applied for *T. pyogenes* phylogenetic investigation.

The routes of *T. pyogenes* infections are frequently difficult to establish. The *T. pyogenes* infections develop mainly as a consequence of the mechanical injuries of skin and mucous membranes [48]. Wounds and abrasions of skin are common routes of infection, for example, udder injuries in the case of *T. pyogenes* mastitis, or hoof injuries in the case of interdigital phlegmon. In the case of mucous membranes damage, such as in metritis, the bacteria can invade deeper tissues after endometrial epithelium, disruption during parturition. Another described route of infection is associated with improperly performed surgical procedures, such as castration or intramuscular injection [103]. Bacteria can also invade tissues through micro-injuries of the skin caused by some arthropoda, like flies or ticks, as it has been mentioned above. In deer, behaviour may influence the pathogen transmission and susceptibility to cranial/intracranial abscess infection, which occur more frequently in adult males [125,127]. Interestingly, Belser et al. [54] noted a higher prevalence of *T. pyogenes* along the forehead of the males of white-tailed deer than the females, when the rate of the pathogen carriage on the nasopharyngeal mucosa was the same for both sexes. Therefore, it is suggested that the disease development may be a result of cuts or abrasions arising from antler rubbing on trees or from sparring between males.

## 4. Pathogenesis of *T. pyogenes* Infection

The pathogenesis of microbial infection is determined by a variety of bacterial and host-related factors. In the case of *T. pyogenes*, the pathogenicity is attributed to the determinants, which induce the formation of abscesses, empyemas, and pyogranulomatous lesions. However, pathogen–host interactions in *T. pyogenes* infection are still poorly understood. Some known and putative bacterial factors contribute to the development of *T. pyogenes* infection, but their role in the infection pathogenesis remains insufficiently explained. Moreover, many researchers highlighted a high phenotypic and genotypic diversity among the studied *T. pyogenes* isolates of various origins, also taking into consideration virulence genotypes [13,21,29,30,31]. This finding indicates the genetic heterogeneity in the studied *T. pyogenes* populations. On the other hand, Cohen et al. [124] detected significantly more virulence genes in *T. pyogenes* isolates from populations of white-tailed deer males, in which a high incidence of the intracranial abscess disease was observed, compared with those detected in the isolates from apparently healthy animals in populations in which the disease was not noted. This finding suggests that the essential differences in the pathogenic potential may exist among *T. pyogenes* strains, depending on their origin.

### 4.1. T. pyogenes Virulence

**Figure 2:**
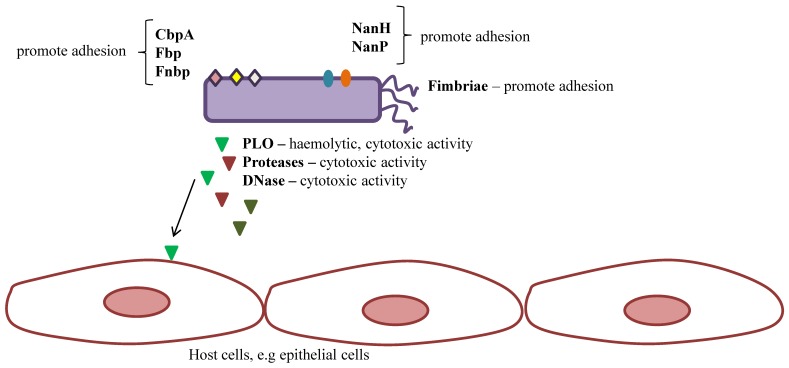
Schematic diagram of known virulence factors of *T. pyogenes*. Abbreviations: CpbA—collagen-binding protein; Fbp—fibrinogen-binding protein; Fnbp—fibronectin-binding protein; NanH—neuraminidase H; NanP—neuraminidase P; PLO—pyolysin.

Only few virulence factors in *T. pyogenes* are recognized to date (Figure 2). They include pyolysin (PLO), the only known toxin of this bacterium; some adhesive factors, such as fimbriae neuraminidases and extracellular matrix-binding proteins; different exoenzymes, such as serine proteases with gelatinase and caseinase activity, or DNAses; as well as the ability to invade host cells and to create biofilm formation [48]. The significance of some of them in the pathogenicity is not clear enough. In general, profiles of genes encoding particular virulence determinants are not correlated with a type of infection and host species. However, a few cases of association between the presence of some determinants and a type of infection were reported [49,55].

#### 4.1.1. Pyolysin

PLO is considered to be both a major virulence factor of *T. pyogenes* and a host-protective antigen [168,169,170], and, until now, the gene (*plo*) encoding this protein was detected in all wild-type *T. pyogenes* strains. This is an exotoxin belonging to the cholesterol-dependent (also called cholesterol-binding) cytolysins (CDCs), which are a family of 51- to 60-kDa single-chain proteins produced by many species of Gram-positive bacteria, for example *Streptococcus pneumoniae* (pneumolysin), *Streptococcus suis* (suilysin), *Streptococcus pyogenes* (streptolysin O), *Paenibacillus alvei* (alveolysin), *Clostridium perfringens* (perfringolysin O), or *Listeria monocytogenes* (listeriolysin O). [171,172]. PLO is the most divergent protein of this group, presenting only 38% to 45 % identity and 58% to 64% similarity to other toxins from the CDC family, when the conserved core of the CDCs (corresponding to amino acid fragment 38–500 of perfringolysin O) is taken into consideration during the analysis [172]. The highest identity of PLO is shown to suilysin, novyilysin, botulinolysin B, and tetanolysin O, which range from 45% to 44% [172]. This protein was purified and characterized for the first time by Ding and Lämmler in 1996 [173], however, its toxic and haemolytic activities were previously reported by Lovell [174]. As a member of the CDC family, PLO displays a cytotoxic effect on a variety of host cells, for example, erythrocytes, polymorphonuclear neutrophils (PMNs), macrophages, epithelial cells, fibroblasts, and endometrial stromal cells. [68,168,171,172]. The cytolytic activity of PLO is associated with its ability to bind to the plasma membrane and to form transmembrane pores, which is a common feature of CDCs [168,172]. The in vitro study showed that PLO is able to cause the lysis of human, sheep, horse, rabbit, and guinea pig erythrocytes [4]. Specific antibodies against PLO completely neutralize its haemolytic activity, suggesting that PLO is the sole haemolysin produced by *T. pyogenes* [168].

The significance of PLO as a primary virulence factor of *T. pyogenes* was confirmed in many in vitro and in vivo experiments, with use of PLO-deficient mutants or recombinant proteins [168,175,176]. PLO has a lethal effect on mice and rabbits, and a dermonecrotic effect on guinea pigs after intravenous and intraperitoneal injection [168,174,177,178,179]. Jost et al. [168] observed a loss of haemolytic activity in the PLO-1 mutant strain as a result of the insertional inactivation of the plo gene, as well as its 1.8-log_10_ reduction in virulence for mice in comparison to the wild-type strain. They also, for the first time, documented the importance of PLO for the in vivo survival of bacteria, studying the effect of the co-challenge of a wild-type strain, and a mutant strain with the inactivated *plo* gene in a mouse model. Similarly, Zhao et al. [179] demonstrated that the *T. pyogenes* strain with an in vitro higher expression of the *plo* gene was more virulent for mice in the in vivo study than the strain with a lower *plo* expression, designated as avirulent.

**Figure 3:**
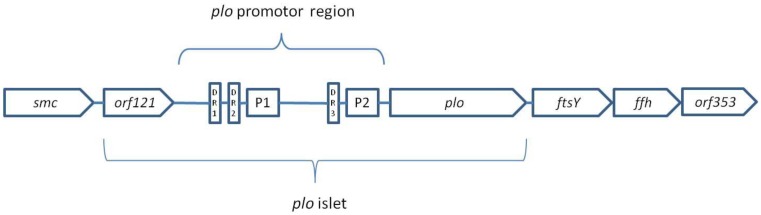
Schematic presentation of the *T. pyogenes* chromosomal region containing the *plo* gene and the surrounding genes. P1 and P2 indicate the positions of two promoters; DR 1–3 indicate the positions of three direct repeats. The scale is not designated.

Billington et al. [180] cloned and sequenced the *plo* gene encoding PLO, which was located in an open reading frame (ORF) of 1605 bp in the *T. pyogenes* chromosom. The *plo* gene sequence was also described by Ikegami et al. [181], for which the deduced pyolysin sequence showed a 99.4% similarity and a 97.6% identity to the PLO sequence previously reported by Billington et al. [180]. A consensus ribosome binding site, two promoter sequences (similar to the *E. coli* σ^70^ promoter), and three direct repeats (DR1 to D3 with sequence ATTTTTG(C)TGG) were found upstream of the *plo* gene, and a transcriptional terminator region was found downstream of this gene [180]. Moreover, Rudnick et al. [170] found that the *plo* gene, along with the mentioned sequences and ORF (*orf121*), encoded a 13.4 kDa protein of an unknown function, form a genomic islet of 2.7 kb, characterized by a reduced content of G+C (50.2%). This islet, flanked by two housekeeping genes, *smc* and *ftsY* (62.5% G+C), is located in the *T. pyogenes* chromosome (Figure 3) [170]. Interestingly, the codon usage of *plo* and *orf121* differ from that of the genes flanking the islet. The genes *smc* and *ftsY* are common in many other bacteria, mainly Gram-positive, and are essential for *T. pyogenes* viability [170]. Downstream of the *ftsY* gene are the *ffh* gene, and then the ORF, *orf353*. The *ftsY* and *ffh* genes encode the FtsY and Ffh proteins, respectively, which have a high similarity to the signal recognition particles from the other bacterial species [170]. The *ftsY*, *ffh*, and *orf353* genes in *T. pyogenes* are located in close proximity, and form an operon-like arrangement that is unusual in most other bacteria. It seems that, as in other species, these genes are needed for bacterial growth.

The differences between the islet and the flanking genes suggest a possibility of horizontal transfer of the *plo* gene, though any integrase or transposon sequences were not found in this chromosomal region. Taking into consideration the fact that the *plo* gene is present in all wild-type *T. pyogenes* strains, including commensal isolates, it seems probable that *plo* was inserted into the intergenic region between *smc* and *ftsY* by homologous recombination; it has to be retained, as both of these genes are obligatorily required for bacterial growth. In addition, it was confirmed by Rudnick et al. [170] that the *plo* islet is a conserved region of the *T. pyogenes* chromosome found in all of the studied isolates of various origins.

*T. pyogenes* is an opportunistic pathogen that, being a part of the host microbiota, has no damaging effect on its organism. Therefore, it is suggested that the regulation of the PLO expression may be critical for the establishment of the status of this bacterium as a pathogen or a commensal. Rudnick et al. [170] reported that the *plo* gene expression is not regulated in vitro by *orf121* located upstream of this gene. In their subsequent investigation, two promoters, P1 and P2, located in the *plo* islet, were identified as the regulatory sequences controlling the transcription of *plo* [57]. It was observed that the in vitro haemolytic activity of *T. pyogenes* was in growth phase depending on a peak in the early stationary phase [57,173]. It indicated that *plo* is up-regulated during that phase, and indeed a significant increase in the *plo* specific mRNA was noted. Thus, the suggestion that the PLO expression is controlled at the transcriptional level was confirmed [57]. It was also shown that the P2 promoter is predominant and highly active during the stationary phase of the in vitro growth of bacteria, while the P1 activity is weak. Besides the P1 and P2 promoters, direct repeats (DRs) located in the *plo* promoter region may be involved in the regulation of the *plo* gene transcription. The study of Rudnick et al. [57] showed that DRs, especially DR3, may function as binding sites for a soluble *T. pyogenes* factor being a transcriptional regulator. However, regarding AT-rich sequences of DRs, which can easily bend, it was supposed that DRs may also act as activators of transcription, by promoting RNA polymerase binding to the promoter.

The predicted, on the basis of the *plo* gene sequence, length of the PLO molecule is 534 amino acids (aa), and then, this protein should have a molecular mass of 57.9 kDa [180]. A signal peptidase cleavage site was found between 27 and 28 aa, indicating that the mature PLO molecule has a weight of 55.1 kDa. According to the Funk et al. [182] study, PLO is an oxygen-stable protein, heat-labile (destroyed at 56 and 100 °C), destroyed by pH 3 and 11, and id sensitive to treatment of protease, trypsin, and amylase.

In contrast to the majority of CDCs, the PLO activity is insensitive to thiols or other reducing agents, in other words, PLO does not require thiol activation [180]. This difference is associated with the divergence within the amino acid sequence of undecapeptide (491–501 aa) of PLO. The undecapeptide is a highly conserved region of each CDC, located near the C terminus of the protein. In some CDCs, including PLO, amino acid substitutions occur in this region [180,183]. In case of PLO undecapeptide (EATGLAWDPWW), a unique cysteine residue (C_491_), responsible for the thiol-activated nature of CDCs, is replaced with an alanine (A_492_). This substitution has no essential effect on the haemolytic activity of PLO, but is related to the oxygen stability of this cytotoxin [184]. Moreover, other changes in the PLO undecapeptide were noted, such as the insertion of a proline residue (P_499_) and the deletion of an arginine residue at the end of the CDCs undecapeptide. It seems that these changes have important effects on the conformation and charge of the undecapeptide [180]. The proline insertion has the greatest effect on the PLO activity, as the deletion of this amino acid resulted in a lack of haemolytic activity [183]. The same effect on the PLO haemolytic activity was observed in the case of the removal or substitution of any of the three tryptophan residues [183]. Therefore, as it was previously demonstrated by Billington et al. [180], the amino acid sequence of the PLO undecapeptide is required for full cytolytic activity.

The cytolytic activity of PLO, like other CDCs, is suppressed by free cholesterol in a concentration of 1µg/mL [180]. However, the presence of cholesterol in the target plasma membrane is absolutely necessary for pore formation by CDCs. The role of the undecapeptide in the initial plasma membrane binding seems to be crucial, in particular, the first tryptophan residue (W_497_) is important. Although, Billington et al. [183] suggested that other fragments of PLO might also participate in recognition and binding to the host cell membranes.

**Figure 4:**
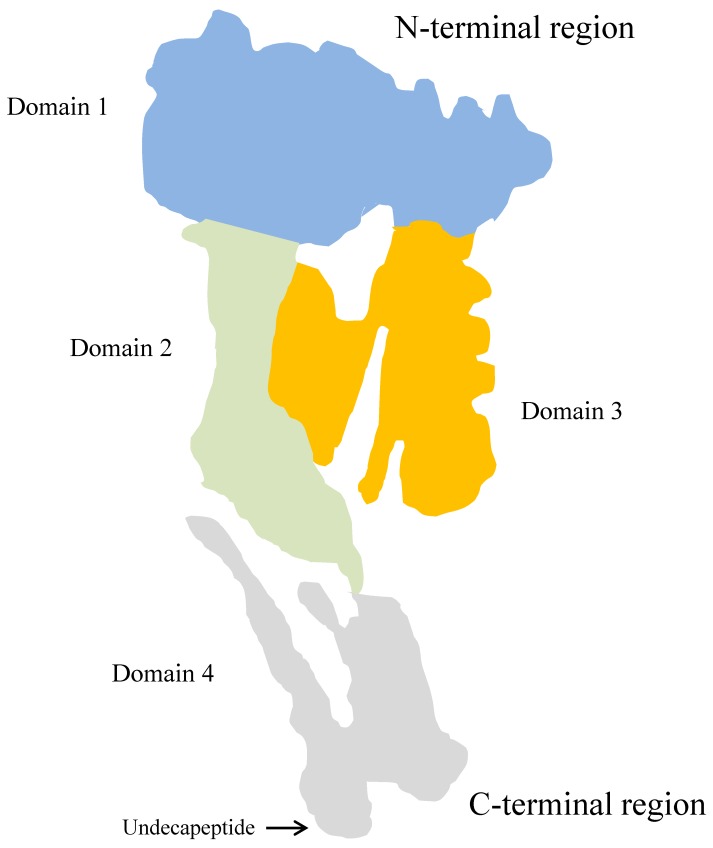
Simplified model of the pyolysin molecule, based on the perfringolysin O-structure.

The known secondary and tertiary structures of some CDCs shows a significant similarity [172]. A crystal structure of the PLO molecule presumably is homologous to the structure of other CDCs. The tertiary structure model of PLO, based on the perfringolysin O (PFO)—PLO sequence alignment—was proposed by Pokrajec et al. [185]. The monomeric PLO molecule is rich in β-sheet elements and consists of four domains (D1 to D4), of which D2 and D3 are packed against each other, while D1 is located at the *N*-terminal and D4 at the C-terminal end of the molecule (Figure 4).

Domain 1 contains a-helix and β-sheet elements. The precise role of D1 in the pore-forming process remains undefined. Zhang et al. [186], studying an effect of the replacement of aspartic acid (D_238_) in this domain with arginine, showed an important role of D1 in maintaining the pore-forming activity of PLO. The investigation of Imaizumi et al. [177] indicated that domain 1, especially the region of 55–74 amino acids, is required for the haemolytic activity of PLO. Yan et al. [187] also confirmed the importance of this region of domain 1, as they demonstrated that the substitution of each amino acid from 58 to 62 in the PLO molecule, particularly isoleucine 61, resulted in the complete loss of its haemolytic activity. Moreover, six B-cell linear epitopes in D1 were identified by Yang et al. [188].

Domain 2 of the molecule forms a long, curved single layer of anti-parallel β-sheet, while the structure of domain 3 is more complex and consists of five strands of anti-parallel β-sheet surrounded by helical layers and short α-helices at either side of the central β-sheet. Domains 2 and 3 are involved in the monomer oligomerization and insertion into the plasma membrane. The separation of D2 and D3, as well as the structural collapse of D2, are required during these processes [189]. The conversion of the D3 short α-helices to two transmembrane β-hairpines starts the molecule insertion into the bilayer, and is a crucial step of the transmembrane β-barrel formation [172].

Domain 4 of the PLO molecule is the only contiguous domain characterized by a β-sandwich structure, connected by a single peptide with the rest of the protein. As it has been mentioned above, the conserved undecapeptide sequence, rich in tryptophan residues, is located at the tip of this domain. This part of the PLO is responsible for host plasma membrane recognition and cholesterol binding [185,190]. However, Pokrajac et al. [185] also demonstrated the ability of D4 to self-oligomerization, but not pore formation. Their findings indicate a greater functional role of this PLO domain in cytolysin activity, compared with the previously described CDCs.

The molecular mechanism of pore-forming by PLO and other CDCs seems to be very similar, and is best described for PFO [172,185]. PLO molecules are secreted to the extracellular environment, probably via the general secretion system, as in the most CDCs [172]. In a form of water-soluble monomers, they bind to the cholesterol-containing areas in the plasma membrane of the eukaryotic host cells [183]. Binding to cholesterol by the PLO domain 4 initiates the oligomerization of the monomers and the formation of incomplete arc-shaped structures, called th pre-pore. Subsequently, the PLO oligomers are inserted into the bilayer by the transmembrane β-hairpins of domain 3, which leads to the formation of a large transmembrane β-barrel pore, which is <50 nm in diameter and protrudes <7 nm above the plasma membrane surface [191]. As a consequence, this process leads to th eleakage of ions and other cytoplasmatic molecules, and finally to the lysis of the host cell.

It seems that a predominant assignment of the cytolytic activity of PLO is to enable access to free iron and other growth factors essential for the bacterial replication contained in the host cells. Furthermore, the ability to lysis phagocytes protects the bacteria against the host immune response. On the other hand, at lower concentrations, PLO can modulate the host immune response.

Despite a wide knowledge about the molecular structure and function of PLO, the precise role of this toxin in the pathogenesis of particular *T. pyogenes* infections, characterized by different course and clinical manifestations, remains unclear.

#### 4.1.2. Fimbriae

The occurrence of fimbriae in Gram-positive bacteria is a rarity. *T. pyogenes* is just one of the exceptions [48]. *T. pyogenes* fimbriae have a filamentous structure, at 200–700 nm in length and 2.5–4.5 nm in width, and probably occur in a limited number on bacterial cells (less than 10 per cell) [48,192]. In *T. pyogenes*, five fimbriae were described, FimA, FimB, FimC, FimE, and FimG [48,192]. The presence of other fimbrial subunits can be deduced on the base of the *T. pyogenes* genome sequences, for example FimJ [192,193]. FimA, 45.7 kDa protein, contains a signal peptide, an E box sequence, and is encoded by the *fimA* gene [48]. FimB, 90.5 kDa protein, has a similar structure, but additionally contains a cell wall-sorting domain and a fibronectin-binding domain, and is encoded by the *fimB* gene [48]. Both genes, *fimA* and *fimB*, are located in a fimbrial gene operon, together with the *srtA* gene encoding a putative sortase. The other fimbriae, FimC, FimE, and FimG, are encoded by the genes *fimC*, *fimE*, and *fimG*, respectively.

The expression of the fimbriae in vitro is poor; therefore, it is difficult to characterize their properties, thus only their genes or mRNAs are better known. Liu et al. [192] investigated the in vitro expression of FimA, FimC, and FimE. However, even though different culture conditions were used, only the FimE expression could be detected. Zhao et al. [179] studied the expression of the virulence factors of *T. pyogenes*, including FimA and FimC, in vitro and in vivo on a mouse model. Their results showed that in in vivo conditions, the *fimA* gene was earlier expressed than *fimC*, which suggests that FimA is a dominating fimbria in *T. pyogenes*. This is in accordance with the findings of many studies on fimbrial genes distribution that demonstrated a high prevalence of *fimA* among various isolates, whereas the genes of other fimbriae were detected with different frequencies [13,21,29,30,31].

A role of fimbriae in the *T. pyogenes* infection is associated with the bacterial adherence to host cells, but the precise mechanisms of this interaction are not well understood. Taking into consideration that *T. pyogenes* can be a commensal as well as a pathogen, it seems that fimbriae are equally important for bacteria in both of these forms. Likewise, in the case of infections caused by many other bacteria, *T. pyogenes* fimbriae are required for cell adhesion and the colonization of host tissues [49,55]. Liu et al. [192], based on their study results, speculated that the poor expression of FimA can limit the formation of other fimbriae, and that the up-regulation of this fimbria production may be associated with *T. pyogenes* pathogenicity.

#### 4.1.3. Extracellular Matrix-Binding Proteins

*T. pyogenes* also expresses other proteins, which determine the adhesive properties, and which are associated with the cell wall of the bacteria. These proteins probably also promote adhesion and enhance colonisation through the ability to bind extracellular matrix (ECM) compounds [48]. In the case of Gram-positive bacteria, these proteins belong to the microbial surface components recognizing the adhesive matrix molecules (MSCRAMM) family [194]. Firstly, *T. pyogenes* shows a fibrinogen-binding activity, however, it was inhibited by proteases [195]. In addition, the 20 kDa fibronectin-binding protein associated with the *T. pyogenes* cell wall was also detected, but not well characterized [48].

Collagen-binding protein A (CbpA) represents the MSCRAMM family, and is one of the better known extracellular matrix-binding proteins produced by *T. pyogenes* [48,194,196]. This 121.9 kDa surface protein is encoded by the chromosomal *cbpA* gene, and displays a 50.4% similarity to Cna, the collagen adhesin of *Staphylococcus aureus*. CbpA binds almost all types of collagen, but not other ECM. However, Pietrocola et al. [194] reported that CbpA can bind fibronectin, but in a distinct subsite than that recognized by collagen. This protein is involved in the adherence of bacteria to epithelial and fibroblast cell lines [196]. It may be supposed that the *T. pyogenes* strain, which produces CbpA, has higher potential to colonize collagen-rich tissues. Although, the *cbpA* gene was found in many isolates of various origins, with different frequencies [13,21,29,30,31].

#### 4.1.4. Neuraminidases

Neuraminidases (sialidases) are one of the most important enzymes of the sialic acid catabolism. Neuraminidases cleave the terminal sialic acid residues from complex glycoproteins, glycolipids, and carbohydrates from the host cell receptors. Neuraminidases are produced by many species of Gram-negative and Gram-positive bacteria or viruses [197]. Bacterial neuraminidases are the main factors for promoting adhesion by exposing the cryptic host cell receptors. In addition, neuraminidases can promote tissue colonisation by reducing mucus viscosity. These compounds also weaken the host’s immune response by exposing IgA particles, making them more susceptible to bacterial proteases [48,197,198,199]. Bacterial neuraminidases are secreted as extracellular enzymes or are anchored to the cell wall. While in Gram-negative bacteria, neuraminidases are usually secreted, neuraminidases of the Gram-positive bacteria are commonly in a cell-associated form [200,201,202,203,204].

The first note about the 50 kDa extracellular neuraminidase of *T. pyogenes* was reported by Schaufuss and Lämmler [205]. Afterwards, two neuraminidases expressed by *T. pyogenes* were better characterized [206,207]. Neuraminidase H (NanH) and neuraminidase P (NanP) are 107 kDa and 186,8 kDa proteins, respectively. These neuraminidases are attached to the *T. pyogenes* cell wall, which results from the presence of specific anchoring motifs in their molecules (LPxTG—for NanH; LAWTG—for NanP). NanH and NanP proteins contain the conserved catalytic RIP/RLP motif (Arg-Ile/Leu-Pro) and five copies of the Asp box motif (Ser-X-Asp-X-Gly-X-Thr-Trp), commonly occurring in the bacterial neuraminidases [206,207]. NanH is the most similar to the neuraminidase from *Actinomyces viscosus* (61.8% similarity and 31.2% identity) [206]. NanP shows a similarity to the neuraminidase from *Micromonospora viridifaciens* (61.6% similarity and 45.3% identity) [207]. However, some similarity between NanH and NanP (53.8% similarity and 38.8% identity) was also demonstrated [48]. 

*T. pyogenes* strains can produce neuraminidases H and P, encoded by the *nan*H and *nan*P genes, respectively [206,207]. The occurrence of these genes is characteristic of the majority of *T. pyogenes* isolates from cattle [21,29,30,31,36,206,207,208] and swine [29,30,36]. However, the *nan*H and *nan*P genes were found at high rates in the bovine isolates studied by Zastempowska and Lassa [37]. The genes encoding neuraminidases are detected with various frequencies in the *T. pyogenes* isolates from small ruminants, such as goats and sheep [29,30,31,36], and in the forest musk deer isolates [134], while the presence of these genes among the isolates from European bison is much less common [29,135]. In several studies, the *nan*P gene was detected more frequently than the *nan*H gene in the tested isolates [30,135,208]. However, Rogovskyy et al. [31] reported that most of *T. pyogenes* isolates from small ruminants carried mainly the *nanH* gene.

*T. pyogenes* neuraminidases play an important role in adhesion to the host cells, as it was shown in the study with epithelial cells [207]. Moreover, *T. pyogenes* neuraminidases are probably necessary for the colonization of host tissues during the early phase of infection, but they are not essential during further stages of a disease development [48]. The different prevalence of the genes encoding neuraminidases in the *T. pyogenes* isolates from various origins, suggests that other adhesion factors may be also produced by this species.

#### 4.1.5. Biofilm

The ability to perform biofilm formation is a well-known feature of microorganisms, which promotes their resistance to many disadvantageous factors, for instance antimicrobials, as well as increases the adherence properties and enables better protection against the immune response of the host [48]. *T. pyogenes*, as with many other bacteria that are resident on the mucous membranes of a host, is able to perform biofilm formation. Biofilm production was noted in a majority of the studied *T. pyogenes* isolates from different animal species and various types of infections [43,134]. Biofilm formation by *T. pyogenes* is controlled by a two-component regulatory system PloS/PloR, where PloR up-regulates the expression of biofilm [64,179,209]. In the case of polymicrobial infection, a biofilm formation by *T. pyogenes* may be inhibited by the QS molecules produced by other bacteria, for example *E. coli* [64,210]. As in some cases biofilm production may promote the infection development, it seems important to find a therapeutical option for reducing this bacterial property. Da Silva Duarte et al. [211] evaluated the use of the *E. coli* phage UFV13 for the disruption of the *T. pyogenes* biofilm, and they obtained a significant decrease of biofilm formation caused by this phage. This finding indicates a necessity of further studies on this issue.

#### 4.1.6. Regulation of Virulence Factor Expression

Jost and Billington [48] suggested the existence of a two-component system comprising a sensor histidine kinase (HK) and response regulator (RR) proteins in *T. pyogenes*. These signalling systems are common among bacteria, and they enable the detection of an environmental or cellular signal that leads to an appropriate cellular response [212,213]. Zhao et al. [64] determined a LuxR-type two-component regulatory system in *T. pyogenes*, named PloS/PloR, using a comparative transcriptome analysis between two *T. pyogenes* isolates from musk deer with different haemolytic activities. The results of the transcriptome analysis showed the presence of ten typical DNA-binding response regulators (RRs) in the *T. pyogenes* genome. However, a protein crystal structure analysis revealed that only one among the identified DNA-binding RRs had a structure similar to that described previously by Jost and Billington [48]. This RR, called PloR, had a CheY-type receiver domain and a C-terminal LuxR-type HTH DNA-binding domain, while a cognate sensor HK was named PloS. Zhao et al. [64] demonstrated that the expression level of the *ploR* gene is correlated with the expression level of the *plo* gene, indicating PloR as an activator of *plo*, which up-regulates PLO production. On the other hand, it was observed that PloR can down-regulate the expression of *T. pyogenes* proteases [48]. In addition, it seems that PloR is able to up-regulate the biofilm formation and probably the expression of the fimbriae in *T. pyogenes*.

The function of the two-component system in *T. pyogenes* was also investigated in vitro using the *N*-acyl homoserine lactones of *P. aeruginosa* and *E. coli* as QS signal molecules, which can bind to the upstream sensor HK, PloS, and via the PloS/PloR system, may regulate the virulence of bacteria by the inhibition of their growth and biofilm production [64]. Huang et al. [65], studying an effect of *N*-acyl homoserine lactones on the *T. pyogenes* virulence in a mouse model, confirmed that those QS molecules inhibited the expression of *plo*, *ploR*, and *ploS*, and increased the survival rate of mice. In conclusion, the function of the two-component system PloS/PloR and the QS system seems to have the essential significance for the modulation of the *T. pyogenes* virulence.

### 4.2. Induction of Host Defense Mechanisms by T. pyogenes

The pathogenicity of *T. pyogenes* is assigned mainly to PLO, a cholesterol-dependent cytolysin, which promotes cell lysis and is responsible for altering the host cytokine profile. This toxin also exerts cytolytic, dermonecrotic, and lethal effects on numerous types of cells, including immunocompetent and non-immune cells [176,186]. It is responsible for the complement cascade induction; modulation of the host cytokine profile; inhibition of respiratory burst; and bactericidal activities in neutrophils, monocytes, and macrophages. These features lead to the modulation of the immune response and to evasion from a host’s immune mechanisms. Because of the presence of neuraminidases NanH and NanP, *T. pyogenes* is able to adhere to the alimentary and respiratory epithelial mucosa, increasing the mucus viscosity that facilitates bacterial colonization and diminishes the host immune response, including damage to a first line of defence—destruction of IgA by bacterial proteases. Those serine proteases, as it was mentioned above, are involved in the invasion and destruction of tissues [48,196].

For many years, the assessment of PLO involvement in the development of the disease was conducted using in vitro (established cell lines or primary cell cultures) and in vivo (mouse and large animal) models [48,68,69,75,168,186].

**Figure 5:**
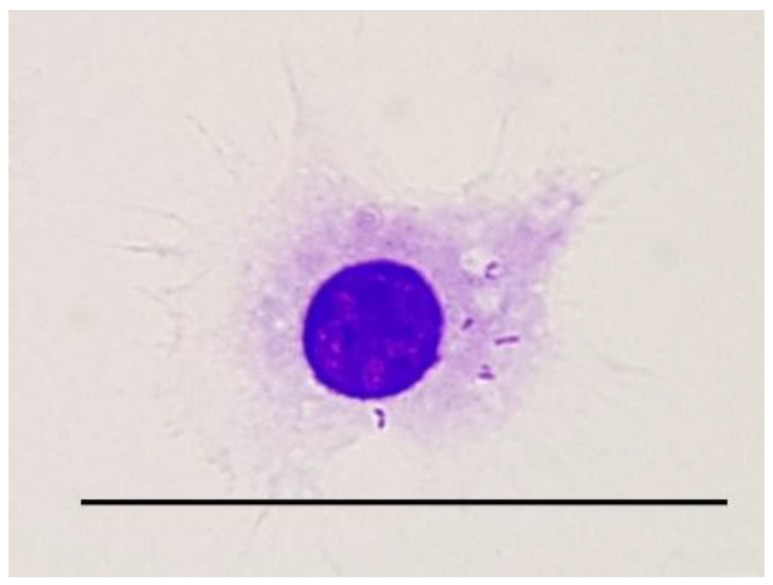
The macrophage of the RAW264.7 cell line infection with *T. pyogenes*. Staining using the Giemsa–MayGrünwald method; scale bar = 20 µm (photo courtesy of Anna Hupało-Sikorska, BSc).

One of the first antibacterial mechanisms of immune response is inflammation, resulting in the recruitment of neutrophils, monocytes, and macrophages to a site of infection. The involvement of those cell populations leads to the phagocytosis and killing of bacteria, clearance of infection, and stimulation of adaptive immune response. Still, *T. pyogenes* interactions with host phagocytic cells strongly influence the outcome of the disease. Using macrophage established cell line J774A.1, it was clearly shown that this pathogen was easily phagocytosed, and could survive within macrophages up to 72 h, with the survival rate diminishing over time. Probably, this is caused by the presence of antibiotics in a medium used for culturing infected macrophages. Once bacteria exit the infected cell, they are killed by the antibiotic present in the medium. Similar results were obtained when the macrophage cell line RAW264.7 was infected with either *T. pyogenes* ATCC 19411 strain or *T. pyogenes* European bison isolate. In this study, phagosome formation, intracellular bacteria survival, and changes in the mitochondrial network and distribution were established at 2, 6, and 24 h post infection (pi) [214]. The formation of phagosomes was noticed as early as 2 h pi, and they were still present within the cells up to 24 h pi, with bacteria either within the phagosome or in the cytoplasm of the infected cells, and the recovery of viable bacteria was possible up to 24 h pi (Figure 5). However, the mitochondrial network morphology and distribution was not visibly altered in the RAW264.7 cells infected with either the ATCC 19411 strain or European bison isolate of *T. pyogenes* when compared with the uninfected control cells. Further investigations are required in order to elucidate the mechanism of *T. pyogenes* pathogenesis. Nonetheless, these data are in agreement with the observations done on bovine polymorphonuclear neutrophils (PMNs) infected with *T. pyogenes*, in which increased phagocytosis was noticed. As this pathogen can survive within phagocytic cells, increased phagocytosis is strongly connected with bacterial dissemination throughout the host [195]. Studies done in a mouse model clearly showed that PLO is responsible for peritonitis [168].

One of the common, most recognized infections in dairy and beef cattle are uterine infections caused by *T. pyogenes*. They result from the recruitment of neutrophils, from monocytes, and from the induction of a proinflammatory response, accompanied with formation of mucopurulent discharges within the infected uterus. This was confirmed on a large animal model. The uterine endometrium of healthy cows was scratched with a cytobrush (damage to the epithelial layer), and subsequently, an intrauterine administration of 5 × 10^8^ cfu of *T. pyogenes* was performed. Mucopurulent uterine discharge was evident in the challenged animals, and was associated with the isolation of this pathogen [68].

However, the ability of cows to defend against *T. pyogenes* infection depends on the interaction between the invading pathogen and the host’s innate immunity. An application of bovine endometrial cell culture in an in vitro model elucidated the PLO involvement in tissue damage and the development of the endometrium pathology of the postpartum period. Recombinant PLO (rPLO) and bacteria-free filtrates (BFF) of *T. pyogenes* had a similar haemolytic activity on the target cells. Still, the stromal cells and epithelial cells expressed a different level of susceptibility concerning treatment with those factors. The endometrial stromal cells were markedly more sensitive than the epithelial cells. This haemolytic effect was abrogated when the endometrial cells were treated with BFF derived from *T. pyogenes* with a deleted *plo* gene (*T. pyogenes*Δ*plo*), and when anti-PLO antibodies were applied. The difference in susceptibility or resistance to PLO between the stromal and epithelial cells can be explained by a lower level of cholesterol expression in the epithelial cells. It has biological implications to PLO-mediated uterine tissue damage. The columnar epithelium of the endometrium establishes a first line of defence by forming a physical barrier between the uterine lumen and the underlying stroma. As it expresses less sensitivity to PLO, it protects the tissue from colonization by *T. pyogenes*. Once this barrier is disrupted, the bacteria can colonize the endometrium, and the development of the disease is induced. However, PLO did not induce inflammation, as the treatment of bovine endometrial culture cells with rPLO did not result in increased levels of IL-1β, IL-6, or CXCL8 in the culture supernatant. Nevertheless, during clinical infection, *T. pyogenes* often causes tissue damage in association with other species of bacteria, expressing together with *T. pyogenes* pathogens, the associated molecular patterns (PAMPs) and damage associated molecular patterns (DAMPs) responsible for the induction of inflammatory response.

The described sensitivity of stromal cells to PLO-mediated cytolysis is one of the explanations for how *T. pyogenes* acts as an opportunistic pathogen, responsible for endometrial damage once the protective epithelium is lost after parturition [68].

It has to be remembered that the development of a disease depends on the ability of an infectious agent to adhere, to colonize a host’s target tissue, and develop mechanism(s) that are responsible for avoiding a host’s defence and the involvement of its innate immunity. In the case of *T. pyogenes*, bovine uterine infections also interplay, as follows: the host–virulent strain of *T. pyogenes* or host–opportunistic strain of *T. pyogenes*, should be considered. The model presented by Ibrahim et al. [56] considers all of these factors. They focused on in vitro investigating the interactions between a bovine endometrial epithelial cell culture with either the *T. pyogenes* strain isolated from the uterus of a postpartum cow developing clinical endometritis, or *T. pyogenes* collected from the uterus of a healthy cow. The assessment of the presence of strain-specific factors participating in the development of the bovine clinical endometritis was also performed. The outcome of the analysis on a genetic level revealed that both strains presented genes encoding virulence factors; however, there were differences between the expression levels of *plo*, *cbpA*, *nanH*, *nanP*, *fimA*, *fimC*, *fimE*, and *fimG* genes, favouring the virulent strain. The proteins encoded by those genes participate in adhesion to the host cells [48,207], so a virulent strain had a higher ability to attach to the epithelium, because of the elevated level of the cell-wall associated molecules. Both strains expressed a cytolytic effect on cultured cells, which means both strains secreted PLO, a major virulence factor. However, this effect was abrogated when the bovine endometrial epithelial cells were treated with heat inactivated bacteria or bacteria-free filtrates. These findings are in line with the results obtained by Amos et al. [68], showing that BFF and rPLO were not responsible for the stimulation of a proinflammatory response in endometrial cells.

**Figure 6:**
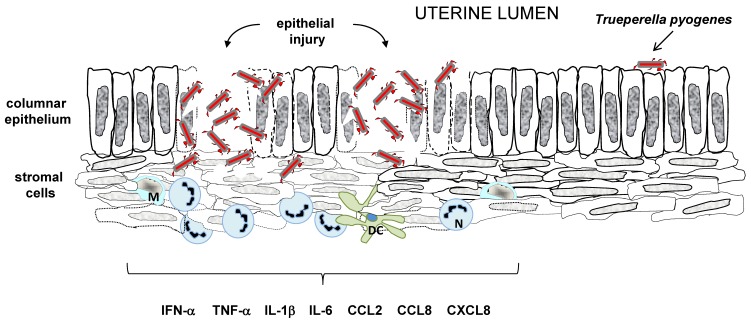
Schematic presentation of the pathogenesis of uterine infection with *T. pyogenes*. Recruitment of monocytes (M), neutrophils (N), dendritic cells (DC), and other proinflammatory cells—secretion of proinflammatory mediators.

To mimic the interplay between the host target tissue and *T. pyogenes* in the chronic proinflammatory environment, endometrial endothelial cells treated with bacteria were co-cultured in the presence/absence of peripheral blood mononuclear cells (PBMCs). The presence of PBMCs did not influence the viability of the endometrial epithelial cells more than 16 h, upon the treatment with live bacteria, irrespective of a strain. However, the early detection (4 h) of genes encoding proinflammatory mediators (*PTGS2*, *CXCL3*, *IL6*, and *CXCL8*) was noticed in the endometrial endothelial cells treated with a virulent strain of *T. pyogenes*, in the presence of PBMCs. This in vitro interaction suggests that the leukocytes found within the endometrium may be responsible for sensitizing the epithelial cells to the initial bacterial infection through the enhancement of uterine innate immunity induction (Figure 6). These data suggest that the development of endometritis in dairy cows after parturition may be caused by a specific characteristic of certain strains of *T. pyogenes*. Moreover, the presence of immune cells can be the reason for the amplification of the proinflammatory response in the endometrial epithelial tissue towards *T. pyogenes* pathogenic strains.

The research of Zhang et al. [186] indicated that the inflammation-inducing effect of PLO depends on its cytolytic activity, but not on PAMPs activity. These findings are in contrast to the results of Amos et al. [68] and Ibrahim et al. [56].

Studies done on bovine oviductal epithelial cells (BOECs), in an in vitro model, suggested a specific mechanism employed by *T. pyogenes* to avoid an immune response. In the course of *T. pyogenes* infection, there was no induction of any clear proinflammatory response in the BOECs at either the transcriptional or protein level. The BOECs co-cultured with *T. pyogenes* remained viable during the first 24 h of incubation when treated with MOI 0.01. However, a higher MOI caused the death of those cells within 24 h of co-culturing. What is interesting, is that the BOECs co-cultured with *T. pyogenes* expressed a similar level of *IL1A*, *IL1B*, and *TNFA* mRNA as the control cells did. Similar results were obtained when the level of mRNA concerning *CXCL8*, *CXCL1/2*, and *PTGS2* was assessed. Although, at the same time, the BOECs co-cultured with *Bacillus pumilus* responded to stimulation, with an increased level of cytokines and chemokines mRNA. It seems that the lack of responsiveness in the BOECs induced by *T. pyogenes* is one of the escape routes from immune surveillance developed by this pathogen, as the expression of the genes encoding the proinflammatory mediators was similar to the control levels [75].

## 5. *T. pyogenes* Genome

The first complete sequence of the *T. pyogenes* genome was described in 2014 by Machado and Bicalho [215]. However, to date, 19 genome assemblies of this species are available in the GenBank nucleotide database (NCBI). Complete genome sequences were reported for 10 strains, isolated as follows: four from swine, three from cattle, one from goat, one from forest musk deer, and one from water buffalo. Additionally, the vast majority of the sequenced genome of *T. pyogenes* were derived from strains, which were isolated from animals in China. Other available genome assemblies are scaffolds (*n* = 3) and contigs (*n* = 6). The main features of complete *T. pyogenes* genomes are summarized in Table 1.

The *T. pyogenes* genome was found to consist of a single circular chromosome comprising from 2.25 to 2.43 mega base pairs, depending on the strain. This species is characterized by high G+C% content. Moreover, the differences in the rate of G+C between the *T. pyogenes* strains are not significant (59.4–59.8%). tRNA is the most abundant RNA type in these bacteria. The share of rRNA in the whole nucleic acid content ranges from three to nine. In addition, there are also several non-coding RNAs (one or three, depending on the strain). The *T. pyogenes* genomes are indicated by the relatively high numbers of pseudogenes (from 27 to 191).

The *T. pyogenes* genome encodes many genes, for which the functions were assigned by the homologies to known proteins. Apart from the virulence-associated genes, the genome of this species can contain several antibiotic resistance genes. Furthermore, the *lux*S gene regulating the formation of biofilms was also found in the genome sequence [209]. In addition, the presence of a member of the *T4virus* (vB_EcoM-UFV13) indicates the efficiency in controlling the biofilm formation by *T. pyogenes* [216]. Moreover, the *T. pyogenes* genome contains four housekeeping genes (*met*G, *tuf*, *gyr*A, and *fus*A). The *tuf* and *fus*A genes encode the translation elongation factors Tu and G, respectively. Two other genes confer the DNA gyrase subunit A (*gyr*A) and methionine—tRNA ligase (*met*G). These genes were successfully used in the multilocus sequence analysis (MLSA) of this species [148]. The genetic analysis showed that *T. pyogenes* had a more complex system of amino acid and lipid metabolism, and more genes involved in the pathogenicity than are found in *Arcanobacterium hemolyticum*. These differences may affect the ability to cause infections in different host types by *T. pyogenes* isolates. Moreover, all of the groups of the family *Actinomycetaceae* have phosphotransferase systems that are probably essential for the colonization of a wide range of hosts and for the initiation of abscess formation. Some genes were lost or acquired as a result of lateral evolution, which helps in the adaptation of bacteria to a new environment [6]. The *T. pyogenes* species was reclassified based on 16S rRNA gene sequences, the menaquinone structure, and the phospholipid composition from the genus *Arcanobacterium* to the genus *Trueperella*, together with four other species, such as *Trueperella abortisuis*, *Trueperella bernardiae*, *Trueperella bonsai*, and *Trueperella bialowiezense*. Furthermore, 16S rRNA gene sequence similarities between strains from the genus *Trueperella* ranged from 95.3% to 98.6% [1]. There was also a draft genome sequence of one human *T. bernardiae* isolate deposited in the NCBI [217].

The genomes of *T. pyogenes* also include plasmids. Until now, two plasmids have been described for this species [178,218]. First, a native pAP1 (U83788) is a circular plasmid of 2439 bp, containing three open reading frames (ORFs). Plasmid pAP1 encodes the *rep* gene, which is required for the initiation of replication. Two other genes, *orf*112 and *orf*129, encode the hypothetical proteins of an unknown function. However, pAP1 does not include any antibiotic resistance genes [218]. The other, pAP2 (AY255627), is a circular plasmid of 9304 bp, and it contains seven ORFs. In pAP2, *rep*A is probably required for the replication of this plasmid. Moreover, there are other genes, namely: *tet*R(33), which encodes the repressor protein and *gcr*Y, and *orf*95 with unknown functions. In addition, pAP2 is characterized by the presence of two genes encoding antibiotic resistance determinants—*erm*(X) and *tet*A(33). In *T. pyogenes*, *erm*(X) determines the resistance to macrolide antibiotics, while *tet*A(33) is associated with low-level tetracycline resistance [178]. Furthermore, plasmid pAP2 contains the insertion sequence IS*6100*, which is found either in Gram-positive or in Gram-negative bacteria [219].

The knowledge of the complete genome sequence may allow for the identification of new genes that may contribute to the pathogenicity and antibiotic resistance of *T. pyogenes*. In addition, genome sequencing can help to understand the basics of the metabolism and evolution of the bacterial species.

## 6. Immunoprotection and Perspectives

The antimicrobial treatment of *T. pyogenes* infections is often ineffective, because of the increasing resistance of bacteria or the limited distribution of drugs to the site of infection, for example, to abscesses. Hence, vaccination should be considered as a primary method of *T. pyogenes* infection prevention. It seems that some of the virulence factors of *T. pyogenes* could be promising candidates for vaccine antigens.

Most studies concerning the stimulation of protective immunity against *T. pyogenes* infection were done on a mouse model, and only a few were done using a ruminant or swine model. The first trials to protect ruminants against infection with *T. pyogenes* were focused on animal treatment with whole cells of *T. pyogenes* or a culture supernatant, and were mostly unsuccessful; however, some therapeutic effects on mastitis development were observed [48,220,221]. In swine, the vaccination of pregnant sows with an autovaccine was an attempt to control the losses of newborn animals. Studies done by Kostro et al. [222] revealed that the administration of *T. pyogenes* cells treated with a phenol solution to pregnant sows, six and three weeks before anticipated delivery, significantly increased the number of CD4^+^ T cells, CD8^+^ T cells, and CD25^+^ T cells, as well as enhanced the levels of IFN-γ, TNF-α, and IL-10 in a colostrum and milk [222]. The recent trials were concentrated on the protection of heifers against post partum uterine diseases. Pregnant heifers were vaccinated using different routes of immunization (subcutaneously and intravaginally), and different compositions of the vaccine, as follows: (i) inactivated bacterial whole cells (*E. coli*, *T. pyogenes*, and *F. necrophorum*) together with recombinant proteins, FimH, PLO, and leukotoxin (LKT)); (ii) recombinant proteins FimH, PLO, and LKT only; (iii) inactivated bacterial whole cells (*E. coli*, *T. pyogenes*, and *F. necrophorum*) only [223]. The obtained results demonstrated that subcutaneous vaccination significantly decreased the incidents of puerperal metritis, but intravaginal vaccination failed in preventing the disease.

Mice used for the assessment of protective immunity induction against infection with *T. pyogenes* provide a reliable model that can serve for further comprehensive studies on other animal species. The administration of formalin-inactivated recombinant PLO (rPLO) induced the protection of mice against intraperitoneal challenge with 10^8^ of *T. pyogenes* cells, and PLO-specific antibodies were detected in the sera of the immunized mice [168,180].

The current trend in vaccine design is concentrated on the development of such a product that will induce immunity against multiple pathogens. Using a mouse model, Zhang et al. [224] and Hu et al. [176] induced specific immunity that protected animals against challenge with *T. pyogenes* and *C. perfringens*. However, their approach to solve the problem concerning the stimulation of protective immunity was quite different. Hu and colleagues [176] designed a chimeric protein called rPC-PD4, which was composed of *C. perfringens* truncated regions of C-domain of phospholipase C (rPLC), and D4 domain of *T. pyogenes* PLO (rPLO), and was encoded by chimeric genes incorporated into a plasmid vector. The production of specific antibodies, and the presence and level of proinflammatory cytokines (TNF-α, IL-1β), chemokines (CXCL8), and IL-10 in sera of immunized mice were assessed; however, only partial immunity was observed after challenge with *T. pyogenes* or *C. perfringens*. Zhang’s group [224] concentrated not only on PLC and PLO as major immunogens, but also on the involvement of formaldehyde inactivated cultures of *T. pyogenes* and *C. perfringens* as vaccine antigens, combined with aluminium hydroxide gel. The outcome of their proposal is promising, as the survival rate of immunized mice after challenge with *T. pyogenes* and *C. perfringens* was 80% and 100%, respectively. These studies [176,224] present a very interesting approach for the development of a new, safe vaccine against polymicrobial infections.

Nonetheless, the latest trials to stimulate immunoprotection against *T. pyogens* infection are the most promising so far. The studies of Huang’s group [225,226] done on a mouse model, concentrated on genetic immunization. Two pathways to stimulate protective immunity against *T. pyogenes* infection were used. The first one applied the modification of the cytokine environment of immunized animals together with delivering the gene encoding PLO [225], the second one focused on immunization with a DNA vaccine containing genes encoding four different virulence factors of *T. pyogenes*, and simultaneous vaccine protection against destruction by the host environment [226].

In the first study, concurrent immunization with two plasmids carrying the *plo* gene and the *IL1β* gene, respectively, resulted in the protection of mice from intraperitoneal challenge, with 3.7 × 10^8^ cfu of *T. pyogenes* [225]. PLO-specific antibodies were detected in the sera of immunized mice with a higher titer of the IgG2a subtype than the IgG1 subtype. The T-cell profile analysis indicated an increased level of CD8^+^ T-cells and CD4^+^ T-cells. These results correlated with the detection and assessment of the cytokine levels evaluated in the supernatant collected from the spleen lymphocytes culture (increased levels of IFN-γ, and IL-2, and slightly diminished IL-4). In this model, IL-1β enhanced the immunogenicity of the vaccine by influencing the activity of the macrophages, the major population of the phagocytic cell, as well as antigen presenting cells (APCs), and also the B-cells, which act as APCs during early phases of immune response development, later transforming into plasma cells secreting antigen-specific antibodies, hence humoral immunity.

In the second research of Huang’s group [226], the *plo*, *cbpA*, *fimA*, and *nanH* genes were paralleled as a single chimeric gene with an attached CpG ODN 1826 motif, so as to overcome low immunogenicity and susceptibility to host endonucleases. Finally, the chimeric gene was incorporated into the expression plasmid vector, and then this construct was encapsulated in chitosan nanoparticles to protect the DNA. The chimeric gene DNA vaccine introduced intramuscularly initiated production of antibodies specific for the target epitopes of PLO, CbpA, NanH, and FimA in mice. Moreover, the level of IgG2a was higher than the IgG1 in the sera of immunized mice, suggesting that immunization with this vaccine mainly primed the Th1 profile.

Compared with the subunit and conventional vaccines, DNA vaccines express some advantages as future stimulants of immunity. They are easy to manufacture, stable, and confer potential safety. Moreover, many genes encoding specific antigen proteins can be inserted in a carrier genome, and once introduced to the host, stimulate protective immunity against a variety of infections. In this context, the studies done by Huang et al. [225,226] are very important, not only in the induction of effective protection against *T. pyogenes* infection. They also proved that the formulated chitosan-CpG ODN nanoparticles are stable in eukaryotic cells, are able to protect the chimeric gene in the encapsulated plasmid from degradation, promote its expression, and also participate in the induction and enhancement of the immune response, so they could serve as a safe and efficient drug release carrier system.

## 7. Conclusions

*T. pyogenes* has significant importance as an opportunistic animal pathogen causing a variety of purulent infections. These infections pose a huge economic problem in livestock breeding. Therefore, it is crucial to understand the mechanisms of pathogenicity of this bacterium and the routes of its transmission for the development of an effective prevention strategy.

Although some of virulence determinants in *T. pyogenes* are well recognized, little is known about the dissemination of infections. It is interesting, there is still limited data about the pathogen–host interactions that may be involved in the development of *T. pyogenes* infection. Especially, the determination of factors that have a key role in transformation from a commensal to a pathogenic bacterium is needed. Although it seems that animals are the main reservoir of this bacterium, the epidemiological relationships between the particular isolates remain unclear.

## Figures and Tables

**Table 1:** General features of the complete *T. pyogenes* genomes available in the GenBank nucleotide database (NCBI).

Strain Designation	Strain Origin	Size (Mb)	GC%	Number of				GenBank Accession nr
				Genes	CDS	RNA	Pseudogenes	
TP4	swine	2.43	59.4	2202	2102	59	41	CP033905.1
TP3	swine	2.38	59.4	2156	2058	58	40	CP033904.1
TP2	cattle	2.25	59.7	2023	1938	58	27	CP033903.1
TP1	cattle	2.33	59.8	2125	1993	58	74	CP033902.1
TP-2849	swine	2.38	59.4	2158	2063	58	37	CP029004.1
TP4479	swine	2.38	59.4	2153	2058	58	37	CP029001.1
Arash114	water buffalo	2.34	59.5	2145	2054	56	35	CP028833.1
2012CQ-ZSH	goat	2.30	59.7	2050	1806	53	191	CP012649.1
TP8	forest musk deer	2.27	59.6	2091	2001	50	40	CP007003.1
TP6375	cattle	2.34	59.5	2082	1984	53	45	CP007519.1
